# Effects of a Powered Ankle-Foot Prosthesis and Physical Therapy on Function for Individuals With Transfemoral Limb Loss: Rationale, Design, and Protocol for a Multisite Clinical Trial

**DOI:** 10.2196/53412

**Published:** 2024-01-26

**Authors:** Jason T Maikos, Alison L Pruziner, Brad D Hendershot, David V Herlihy, John M Chomack, Michael J Hyre, Samuel L Phillips, Alexis N Sidiropoulos, Christopher L Dearth, Leif M Nelson

**Affiliations:** 1 Veterans Affairs New York Harbor Healthcare System New York, NY United States; 2 Walter Reed National Military Medical Center Bethesda, MD United States; 3 Extremity Trauma and Amputation Center of Excellence Falls Church, VA United States; 4 National Veterans Sports Programs and Special Events Department of Veterans Affairs Washington, DC United States; 5 Uniformed Services University of the Health Sciences Bethesda, MD United States; 6 The Narrows Institute for Biomedical Research and Education Brooklyn, NY United States; 7 James A. Haley Veterans' Hospital Tampa, FL United States

**Keywords:** amputation, limb loss, physical therapy, powered prosthetic ankle-foot device, lower extremity

## Abstract

**Background:**

Powered ankle-foot prosthetic devices can generate net positive mechanical work during gait, which mimics the physiological ankle. However, gait deviations can persist in individuals with transfemoral limb loss because of habit or lack of rehabilitation. Prosthetic research efforts favor the design or evaluation of prosthetic componentry and rarely incorporate any type of rehabilitation, despite evidence suggesting that it is critical for minimizing gait imbalances. Given the accelerated rate of innovation in prosthetics, there is a fundamental knowledge gap concerning how individuals with transfemoral limb loss should learn to correctly use powered ankle-foot devices for maximum functional benefit. Because of the recent advances in prosthetic technology, there is also a critical unmet need to develop guidelines for the prescription of advanced prosthetic devices that incorporate both physical and psychological components to identify appropriate candidates for advanced technology.

**Objective:**

The primary goal of this investigation is to examine the roles of advanced prosthetic technology and a device-specific rehabilitative intervention on gait biomechanics, functional efficacy, and pain in individuals with transfemoral limb loss. The secondary goal is to develop preliminary rehabilitation guidelines for advanced lower limb prosthetic devices to minimize gait imbalances and maximize function and to establish preliminary guidelines for powered ankle-foot prosthetic prescription.

**Methods:**

This prospective, multisite study will enroll 30 individuals with unilateral transfemoral limb loss. At baseline, participants will undergo a full gait analysis and assessment of function, neurocognition, cognitive load, subjective preferences, and pain using their current passive prosthesis. The participants will then be fitted with a powered ankle-foot device and randomized into 2 equal groups: a powered device with a device-specific rehabilitation intervention (group A) or a powered device with the current standard of practice (group B). Group A will undergo 4 weeks of device-specific rehabilitation. Group B will receive the current standard of practice, which includes basic device education but no further device-specific rehabilitation. Data collection procedures will then be repeated after 4 weeks and 8 weeks of powered ankle use.

**Results:**

This study was funded in September 2017. Enrollment began in September 2018. Data collection will conclude by March 2024. The initial dissemination of results is expected in August 2024.

**Conclusions:**

The projected trends indicate that the number of individuals with limb loss will dramatically increase in the United States. The absence of effective, evidence-based interventions may make individuals with transfemoral limb loss more susceptible to increased secondary physical conditions and degenerative changes. With this expected growth, considerable resources will be required for prosthetic and rehabilitation services. Identifying potential mechanisms for correcting gait asymmetries, either through advanced prosthetic technology or rehabilitative interventions, can provide a benchmark for understanding the optimal treatment strategies for individuals with transfemoral limb loss.

**Trial Registration:**

ClinicalTrials.gov NCT03625921; https://clinicaltrials.gov/study/NCT03625921

**International Registered Report Identifier (IRRID):**

DERR1-10.2196/53412

## Introduction

### Background

There are approximately 1.9 million Americans with limb loss today, with an estimated 185,000 people who undergo an amputation procedure each year [1]. Over the last 2 decades, the Department of Veterans Affairs (VA) and the Department of Defense (DoD) have experienced an increase in the number of veterans and service members with lower limb loss [2]. Since the start of the most recent conflicts, more than 1700 service members have experienced combat-related limb loss, with the vast majority of these traumatic amputations of the lower limb [2-4]. VA, the largest integrated health care network in the United States, serves this unique population after their separation from active duty and provides care for an additional 41,000 veterans with lower limb loss [5]. Transfemoral limb loss, the second most common level of lower limb loss, accounts for one-fifth of the total limb loss population in the United States [6]. With this already large population expected to grow, effective outcomes-based clinical practice will be necessary to improve mobility, decrease long-term disability, and provide a higher quality of life.

### Abnormal Gait Mechanics for Individuals With Transfemoral Limb Loss

Individuals with transfemoral limb loss have unique functional challenges owing to the loss of the knee and ankle joints [7-9]. Gait mechanics of individuals with transfemoral limb loss have been extensively investigated, with abnormalities typically characterized by asymmetries in stance phase biomechanics [10-13]. Individuals with transfemoral limb loss exhibit increased ipsilateral hip extensor activity and hip power, which is thought to be compensation for the lack of ankle power normally provided by the gastroc-soleus complex [14]. Consequently, compensatory mechanisms at joints proximal to the level of limb loss are often used to replace the function normally delivered by the muscles surrounding the ankle joint [12]. Individuals with unilateral transfemoral limb loss also tend to walk with longer stance times on the intact versus prosthetic limb [10], which can lead to a corresponding asymmetrical load distribution [15]. These asymmetrical joint forces place greater demands on the intact limb, which may explain the higher prevalence of musculoskeletal injuries, pain, and joint degeneration of the intact limb compared with uninjured individuals [16,17]. Significant among these secondary conditions is pain, specifically in the intact knee and lower back. In a study of experienced prosthesis users, knee pain in the intact limb was the primary complaint of 75% of individuals with transfemoral limb loss [18]. In a sample of 63 male veterans with traumatic lower limb loss, individuals with transfemoral limb loss were 5 times more likely to have intact knee pain compared with neurotypical participants [16]. Among individuals with lower limb loss (both transtibial and transfemoral), 71% reported back pain within the previous month, but individuals with transfemoral versus transtibial limb loss were significantly more likely to have greater pain intensity [19]. Chronic, persistent pain can lead to limitations in function. There is a significant need to explore the effects of advanced prosthetic technologies and rehabilitative interventions on pain reduction, function, and biomechanics.

### Biomimetic Prosthetic Technology

New technologies in lower limb prostheses have attempted to combat gait pathologies by generating biomimetic ankle power through spring-clutch mechanisms or advanced sensor and actuator technology [20]. Recent advances in microelectronics, battery technologies, and the development of several new types of actuators [21,22] have ushered in the development of powered lower limb prostheses that can better replicate the positive work phases of the ankle through the use of actuators, motors, or pneumatic muscles [23-27].

The Empower (Ottobock Inc), which uses a series-elastic actuator and a carbon-composite footplate, is currently the only commercially available powered ankle-foot device [28,29]. The Empower has been investigated in the population of individuals with transtibial limb loss, but it has yet to be fully investigated in individuals with transfemoral limb loss [30-33]. In a study of individuals with transtibial limb loss, the use of a powered versus passive ankle-foot device reduced the peak resultant force and knee adduction moment on the unaffected leg during level ground walking, potentially limiting the risk of secondary musculoskeletal comorbidities [31]. Individuals with transtibial limb loss using the same powered ankle-foot device had improved ankle power, greater net trailing limb step-to-step transition work, and a lower metabolic rate compared with a passive energy storing and returning ankle-foot prosthesis during level ground ambulation [32].

Although biomimetic prosthetic devices can better approximate biological ankle biomechanics, residual gait deviations can persist, either because of habit or a lack of proper rehabilitative training. For example, despite greater ankle power generation with powered ankle-foot device use, individuals with transtibial limb loss can still walk with compensatory strategies at the proximal joints, which can be attributed to the introduction of new interlimb asymmetries from the uniarticular function of the device [30]. Therefore, device-specific rehabilitation may be needed to minimize or correct the reported deficiencies. Similarly, in the absence of an evidence-based rehabilitation program to correct or minimize preexisting gait asymmetries, instrumented gait analyses that assess the biomechanical function of prosthetic devices may be more likely to quantify the physical gait deviations developed through habit or lack of training rather than device-specific attributes [34]. Therefore, it may be more accurate to postulate that powered ankle-foot devices, through the generation of normative ankle power during push off, offer an opportunity to minimize gait deviations and normalize prosthetic function but not without the incorporation of a rehabilitation program to train prosthesis users to reduce existing gait deviations.

### Prosthetic Rehabilitation Programs

The current state of prosthetic research efforts appears to favor the design and evaluation of prosthetic componentry, particularly with respect to gait mechanics, and rarely incorporates or reports any type of physical therapy (PT) program or device-specific training [34]. Given the accelerated rate of technological innovation in prosthetic devices, there is a fundamental knowledge gap concerning how individuals with lower limb loss should learn to correctly use this advanced, powered technology for maximum benefit. However, previous investigations have examined the effectiveness of rehabilitation protocols on the outcomes of individuals with transfemoral limb loss who used passive prosthetic devices. Prosthetic gait training based on proprioceptive feedback for individuals with transfemoral limb loss was more effective for improved weight-bearing and temporal-spatial parameters than traditional gait training [35]. Sjodahl et al [36] used instrumented gait analysis to measure the gait parameters of individuals with unilateral transfemoral limb loss before and after a training program and reported improved walking speed and sagittal plane hip kinematic symmetry after training. However, the authors also reported increases in compensatory strategies for the intact limb, including an increase in the intact knee extension moment. Virtual reality–based gait training with real-time biomechanical feedback improved frontal plane hip, pelvis, and trunk motion during level ground walking [37]. Currently, there have been no published studies detailing the effects of a device-specific rehabilitation program on the biomechanical or functional outcomes of individuals with transfemoral limb loss who use a powered ankle-foot prosthesis. In this investigation, this knowledge gap will be addressed, and a benchmark to understand optimal treatment strategies will be provided for individuals with transfemoral limb loss to minimize gait impairments.

### Summary

The development of evidence-based health care practices is critical to maximizing prosthetic and health outcomes in the growing population of individuals with transfemoral limb loss. Identifying potential mechanisms for correcting gait asymmetries, through advanced prosthetic technology, rehabilitative interventions, or both, can provide a benchmark to better understand the optimal treatment strategies for individuals with transfemoral limb loss. Despite research suggesting that an evidence-based rehabilitation program that incorporates prosthetic gait training is a critical factor in minimizing compensatory mechanisms [38-40], most prosthetic device protocols fail to incorporate any type of significant rehabilitation or device-specific training. Therefore, this investigation will be the first to elucidate the effects of an advanced powered prosthesis and the role of rehabilitative interventions on gait biomechanics, performance, and pain in individuals with transfemoral limb loss.

### Study Objectives

The overarching goal of this investigation is to examine the roles of advanced prosthetic technology and a device-specific rehabilitative intervention in individuals with transfemoral limb loss. The central hypothesis is that powered plantarflexion, coupled with an evidence-based, device-specific PT intervention, will improve biomechanical outcomes, which will correlate with decreased pain and improved functional performance. The objectives of this investigation are as follows:

1. To examine the biomechanical and functional efficacy of a powered prosthesis compared with a passive prosthesis for individuals with transfemoral limb loss
2. To determine the effects of a powered prosthetic ankle-foot device and a PT intervention on lower extremity kinematic and kinetic patterns, functional efficacy, and pain in individuals with transfemoral limb loss
3. To develop preliminary rehabilitation guidelines for a powered ankle-foot device to minimize gait imbalances and maximize function, as well as to establish preliminary guidelines for powered ankle-foot prosthetic prescription

## Methods

### Study Overview

**Figure 1:**
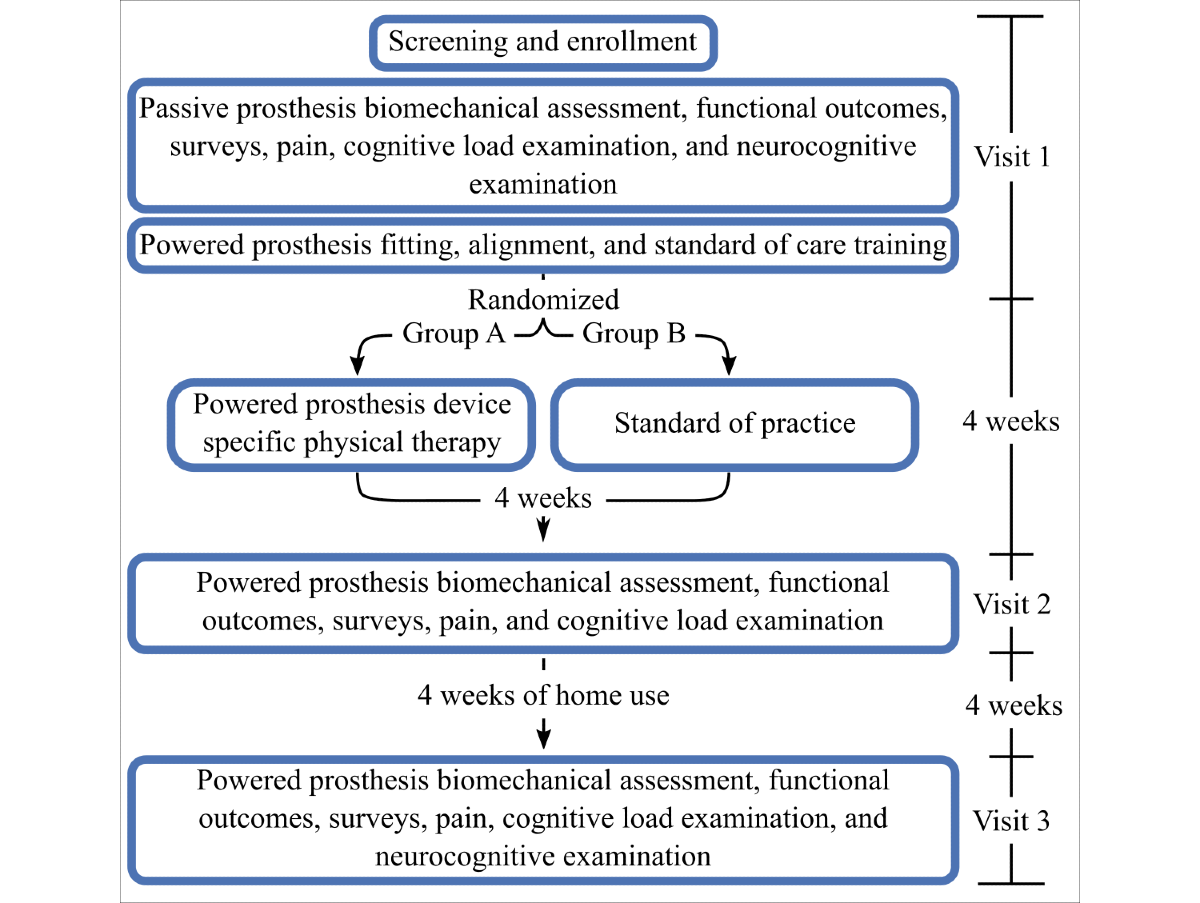
Participant timeline of activities.

This investigation will be a prospective, multisite study including VA New York Harbor Healthcare System (VANYHHS), James A. Haley Veterans’ Hospital (JAHVH), and Walter Reed National Military Medical Center (WRNMMC). Enrollment began in September 2018, and data collection is expected to conclude in 2024. Briefly, 30 individuals with transfemoral limb loss are expected to be enrolled equally across the 3 sites. For all participants, a full biomechanical gait analysis, functional measures, surveys, neurocognitive assessment, cognitive load assessment, and pain assessment will be captured at baseline with their clinically prescribed passive energy storing and returning ankle-foot prosthesis. The participants will be fitted with a powered ankle-foot device (Empower) and then be evaluated for safe use. The participants will then be randomly assigned into 2 groups: a powered ankle-foot device with a 4-week, 8-session device-specific PT intervention (group A) or a powered ankle-foot device with the current standard of practice (group B), which includes basic device education and training provided by the study prosthetist (outlined in the Powered Ankle-Foot Device Standard of Practice section), but no device-specific PT intervention. Group A will then undergo 4 weeks of device-specific rehabilitation, while group B will not receive any further PT. All participants will then undergo a full gait analysis as well as assessments of function, subjective preferences, neurocognition, cognitive load, and pain after 4 weeks and 8 weeks of powered ankle-foot device use (Figure 1). A comparison between the 2 groups will help evaluate the efficacy of a powered versus passive prosthesis, as well as elucidate the contribution of device-specific effects to rehabilitation-specific effects for individuals with transfemoral limb loss.

### Participants

A convenience sample of 30 individuals with unilateral transfemoral limb loss will be recruited for this study (Textbox 1 shows the inclusion and exclusion criteria). All participants will consent to participate before participating in any study activities. The participants will be randomly stratified into 2 study arms: group A, a powered ankle-foot device with device-specific PT (15/30, 50%), and group B, a powered ankle-foot device with standard of practice (15/30, 50%), which includes basic device education and training but no device-specific PT intervention. Recruitment of participants will be on a first-come, first-serve basis among the patients of VANYHHS, JAHVH, and WRNMMC. The participants will be recruited through the VANYHHS, JAHVH, and WRNMMC rehabilitation and prosthetic clinics. All participants will have experience using a microprocessor knee and will currently use a passive-elastic ankle-foot prosthesis.

**Inclusion and exclusion criteria.**

**Inclusion criteria**

- Unilateral transfemoral limb loss because of any etiology
- Use of a microprocessor knee with >6 months of experience
- ≤8 limb loss–related physical therapy sessions in the previous 6 months
- Aged at least 18 years
- Score of ≥33 on the Amputee Mobility Predictor, corresponding to a high K2 or above ambulator
- Able to walk a minimum of 30 meters without an assistive device
- Able to walk on a treadmill for 5 minutes at self-selected speed with or without the use of handrails

**Exclusion criteria**

- Inability to tolerate the wearing of a socket or a poorly fitting socket
- Conditions of the intact limb prohibit prosthesis use (eg, ulcers, sores, skin breakdown, burns, poor skin coverage, contractures, and severe heterotopic ossification)
- The length of the residual limb prohibits socket or prosthesis fitting
- Cognitive deficits or a mental health pathology limiting the ability to participate fully in the study or any deficit deemed by the principal investigator to be detrimental to the completion of the study
- Significant comorbidity, which would interfere with the study (eg, neuropathy, uncontrolled diabetes, receiving dialysis, insensate feet, severe phantom pain, or a history of skin ulcers)
- Severe circulatory problems, including peripheral vascular disease and pitting edema
- Pregnant women in the second trimester or beyond or women who will be in the second trimester within the enrollment period
- Weigh >130 kg at screening
- Use of nonprescribed opioids or overuse of any prescription drugs
- Major upper limb loss
- Currently uses a powered ankle-foot prosthesis as a primary prosthesis or used a powered ankle-foot device as a primary prosthesis in the previous 6 months
- Any cardiopulmonary, metabolic, or integumentary diagnosis where walking for 15 minutes is contraindicated

### Ethical Considerations

This study was approved by the following institutional review boards (IRBs): VANYHHS IRB (1643), WRNMMC IRB (WRNMMC-2018-0167), and the University of South Florida IRB, which is the IRB of record for JAHVH (IRB STUDY000870). The oversight and protection of human participants was also approved by the US Army Medical Research and Development Command Office of Human Research Oversight (E04081). All participants will provide informed consent before participating in any study activities.

### Baseline Visit

#### Informed Consent, Enrollment, and Randomization

Informed consent for each potential participant will be conducted in person in a private room. The site-principal investigator or qualified designee will explain the study protocol in detail. The participants will be asked to consent to randomization of the treatment group, either to be fit with the powered ankle-foot device and receive device-specific PT (group A) or to be fit with the powered ankle-foot device and receive the current standard of practice that does not include device-specific PT (group B). The participants will be asked to make a commitment to be available for all study-related activities. The individuals will be given adequate time to review and comprehend all information about the study before agreeing to participate, minimizing the possibility of coercion and undue influence. After the study has been explained and consent has been given, the participants will be randomized into the 2 groups using a computer-generated algorithm that block randomizes participants into each group at each site.

#### Baseline Data Capture

Regardless of the group assignment, all participants will undergo baseline data capture using their current passive energy storing and returning prosthesis. The study prosthetist will ensure proper fit and alignment of the prosthesis before any data collection. Once fit and alignment are confirmed, the participants will complete 3 surveys: an assessment of quality of life, the Prosthesis Evaluation Questionnaire (PEQ), and the PEQ Addendum. Then, pain, cognitive load, neurocognition, biomechanical gait analysis, and functional outcomes will be captured at baseline for all participants on their current passive energy storing and returning prosthesis.

#### Quality-of-Life Assessment

A single-item assessment will ask the participants to rate their quality of life over the past 4 weeks on a 100-mm visual analog scale.

#### Assessment With PEQ

The PEQ is a self-reported visual analog–style questionnaire for people with lower limb loss who use a prosthesis to evaluate the prosthesis and life with the prosthesis. The PEQ is organized into 9 domains that may be used independently to measure a specific domain of interest. Domains of utility, appearance, sounds, residual limb health, and ambulation will be used in this investigation. In addition, the PEQ contains items beyond the domains that can be evaluated individually, including questions on satisfaction, pain, and transfers that will be used in this study [41].

#### Assessment With PEQ Addendum

The PEQ Addendum asks 2 open-ended questions assessing any falls or stumbles that the participant may have experienced over the previous 4 weeks [42].

#### Pain Assessment

Participants will complete the Patient-Reported Outcomes Measurement Information System Pain Interference Scale 8a, which is a self-report survey that assesses the extent to which pain interferes with physical, psychosocial, cognitive, emotional, and recreational activities [43].

#### Neurocognitive and Cognitive Load for Prosthesis Use

Prosthesis use requires physical capabilities and the cognitive capacity to learn new techniques across different situations and environments. These skills include spatial organization, memory, attention, and visuospatial function [44]. Powered ankle-foot devices can be more complex than passive devices and may require certain levels of neurocognition and cognitive load for ambulation, especially for higher-level functional tasks. Diabetes and peripheral vascular disease, the most prevalent causes of lower limb loss, are linked to declining neurocognition [45,46]. Importantly, diminished neurocognitive function is not often observed until late in the rehabilitation process (well after prosthetic prescription) [47], which can result in the mismanaged use of staff and patient time and prosthetic resources. If certain levels of neurocognitive abilities correlate with successful prosthetic outcomes with the powered prosthesis, neurocognitive assessment (before prosthetic prescription) can potentially aid in the selection of appropriate candidates for advanced technology.

#### Cognitive Load Assessment

Cognitive load assessments will be performed at baseline using the passive energy storing and returning ankle-foot prosthesis. Before any cognitive load testing, a self-selected walking speed will be established via treadmill walking. The participants will be encouraged to walk unsupported on the treadmill, if possible. The treadmill console will be covered to prevent number distractions, and the participants will be reminded to hold their gaze straight ahead. To determine the self-selected speed, the participants will begin walking on the treadmill at a comfortable speed in the absence of any additional cognitive load. The treadmill will then be increased by 0.09 m/s every 10 seconds until the participant verbalizes their preferred speed. To avoid quick trigger responses, the speed will be increased by 0.18 m/s and then subsequently lowered by 0.04 m/s every 5 seconds until the participant verbalizes their preferred speed. If the final speed does not match the initial speed, this procedure will be repeated until the participant matches the preferred walking speed.

Cognitive load testing consists of five 1-minute standardized cognitive tasks: 1 serial subtraction task, 3 controlled oral word association tasks, and 1 category task. These tasks consist of auditory and verbal cognitive measures to simulate real-world conditions. Visual tasks will not be used to avoid measures that require reading while walking. The participants will complete each cognitive task while walking on a treadmill at the previously noted self-selected speed. The directions will be read to each participant to ensure protocol consistency. The number of correct answers will be determined for each 1-minute test using a digital voice recorder and paper recordings to ensure accurate documentation of the participants’ answers. The participants will complete a practice trial before each task initiation to ensure complete comprehension. The participants will also be allowed to rest between cognitive tests as needed. The cognitive tasks are as follows:

- Serial subtraction: Serial subtraction is a mental arithmetic task [48]. The participants are given a random 3-digit number and asked to continually subtract 3 while they walk for 1 minute. The number of errors will be calculated. The participants are not penalized for multiple errors if 1 error was made; however, they continued sequentially thereafter.
- Controlled Oral Word Association Test: This measure consists of 3 tests that measure verbal phonemic fluency and other neuropsychologic domains [49]. The participants will be asked to list words beginning with a certain letter while walking for 1 minute. The test is then repeated for 2 other letters. The total number of unique words for each letter will be documented.
- Category Test: The participants are asked to list words belonging to a certain category within 1 minute (eg, fruits or parts of a car). Category naming has shown validity and reliability [50]. The total number of unique words for each category will be documented.

After each cognitive load test, the participants will be asked to rate on a 0 to 10 scale (with 0 being “none” and 10 being “a great deal”) their focus on walking, their focus on thinking of words or subtracting numbers, and their focus on distractions. This will provide information on the subjective experience of cognitive load.

#### Neurocognitive Assessment

Following the cognitive load assessment, the participants will take an electronic neurocognitive battery (CNS Vital Signs) [51]. The computerized neurocognitive assessment measures 5 domains (memory, psychomotor speed, reaction time, complex attention, and cognitive flexibility), is designed to be administered serially, and has demonstrated good test-retest reliability. The neurocognitive assessment consists of the following 7 tests:

- Verbal memory: the participants are instructed to remember 15 words that are displayed 1 at a time every 2 seconds. The target words are then randomly mixed with 15 new words. The participants are instructed to press the space bar when a target word is displayed. This test is repeated at the end of the assessment with the same 15 target words randomly mixed with 15 new nontarget words.
- Visual memory: the participants are instructed to remember 15 geometric shapes that are shown 1 at a time every 2 seconds. The shapes are then randomly mixed with 15 new nontarget shapes. The participants are instructed to press the space bar when they identify a target shape. This test is repeated at the end of the neurocognitive assessment period.
- Finger tapping: the participants are instructed to tap the space bar with their right index finger as quickly as possible during the 10-second test. The test is then repeated with the left index finger.
- Symbol Digit Coding: this test consists of 8 digit-symbol pairs, followed by a list of digits. The participants are instructed to serially type numbers that correspond to the symbols during the 120-second test.
- Stroop test: in part 1 of the Stroop test, the participants are randomly shown the words “GREEN,” “YELLOW,” “RED,” and “BLUE.” These words are printed in black. The participants are instructed to press the space bar when they see one of these words. In part 2, the same words appear on the screen but are printed in different colors. The participant is instructed to press the space bar when the color presented matches the word (eg, the word “RED” is printed in red). In part 3, the same words are presented on the screen in different colors. The participants are then instructed to press the space bar when the color presented does not match the word (eg, the word “RED” is printed in green).
- Shifting Attention: in this test, 3 figures appear on the screen. There is a single figure at the top of the screen (either a circle or a square). At the bottom of the screen, 2 figures are presented (both a square and a circle). The figures are randomly mixed to be either red or blue. The participant is then instructed to match one of the corresponding bottom figures with the top figure by shape or color. However, the rules for matching by shape or color change at random. This test occurs for 90 seconds.
- Continuous performance test: during a 5-minute period, the participants are asked to press the spacebar when the letter *B* appears on the screen. The participants are further instructed not to respond to any other letters. The letters are presented at random.

#### Biomechanical Gait Analysis

Gait analyses will be performed at the biomechanics laboratories at VANYHHS, WRNMMC, and JAHVH. The VANYHHS laboratory is a 133-m^2^ space comprising an 11-camera motion capture system (Qualisys, Inc) with 4 multiaxis force platforms (AMTI, Inc). At WRNMMC, the biomechanics laboratory is an 167-m^2^ space comprising an 18-camera motion capture system (Qualisys, Inc) and 6 multiaxis force platforms (AMTI Inc). At JAHVH, the motion capture laboratory is a 74-m^2^ space equipped with a 12-camera motion capture system (Vicon Inc) and 4 multiaxis force platforms (AMTI Inc). All 3 systems track the positions of passive reflective markers at a rate of 120 Hz, and force platforms sample ground reaction forces at a rate of 1200 Hz. Visual3D software (C-Motion Inc) will be used for the analysis of 3D motion capture data.

All laboratories in this investigation will follow recommendations provided by an interlab reliability study that was conducted between gait laboratories at the 3 military treatment facilities [52]. Specifically, all sites will use identical marker sets, identical anatomical segment definitions, and a single examiner at each site to conduct postprocessing of the respective data to reduce potential variability between the laboratories.

For all participants, biomechanical gait analysis will be performed at baseline using their prescribed energy storing and returning prostheses. All kinematic and kinetic biomechanical measures will be captured using an identical 6-degrees-of-freedom marker set. A custom, full-body passive reflective marker set will be placed on each participant, which tracks each segment independently, allowing for the accurate measurement of movements. As previously described [53], 78 markers will be placed or digitized on the head, trunk, pelvis, and extremities. Marker placements for the prosthetic limb will be matched to those of the intact leg or placed on the centers of rotation of the prosthetic ankle-foot and knee devices. The cluster technique will be used to minimize the surface-to-bone displacements for the thigh, shank, and upper arm–mounted markers [54]. Tracking clusters will be placed bilaterally on the thigh, the tibial crest, and the upper arm. Functional joints, adapted from Schwartz and Rozumalski [55], will also be calculated for the intact ankle and knee as well as bilaterally for the hips.

During each experimental session, the participants will separately walk at 3 speeds across an instrumented walkway until 5 acceptable trials for each foot at each speed are completed. Trials will be considered acceptable when a foot makes full contact with a force platform. Because kinetic outcome measures are speed-dependent, the participants will ambulate at 3 controlled speeds: 0.7, 1.0, and 1.3 m/s. These speeds were selected to represent a slow, moderate, and fast walking speed, respectively, for individuals with transfemoral limb loss. The order of speeds will be randomized for each data collection visit. Auditory feedback will be provided to the participant by the study team to ensure that all participants walk at the targeted speed (−5% to +5%). The main purpose of this session is to collect joint motion, force, torque, and power data at each walking speed. The ranges of motion; speeds and accelerations; and hip, knee, and ankle joint moments of force and generated and absorbed powers will be computed using inverse dynamics methods. Temporal-spatial parameters will also be recorded.

The reflective marker positions will be digitized using motion tracking software. A 15-segment rigid body model (head, trunk, pelvis, bilateral upper and lower arms, hands, thighs, shanks, and feet) will be created based on the skin-mounted markers and functional joints. Local coordinate systems for each segment will be defined using the International Society of Biomechanics recommendations [56,57]. The data of 5 acceptable walking trials at each speed will be processed using Visual3D. Marker data will be filtered with a 6 Hz Butterworth low-pass filter. Raw analog data will be filtered using a second-order low-pass Butterworth filter with a 25-Hz cutoff frequency. Visual3D will be used to calculate temporal-spatial values, walking speed, and lower extremity kinematics and kinetics. Inverse dynamic analysis will be applied to the kinematics of the biomechanical model and to the location, magnitude, and direction of ground reaction forces acting on the foot to calculate lower extremity joint torques and powers, including ankle, knee, and hip power of the biological and prosthetic limb over the stance phase, as well as the frontal plane knee moments for the unaffected leg.

#### Functional Outcome Measures

The effects of the prosthetic devices and the rehabilitative intervention on physical performance will be evaluated using agility and mobility tests, including the 6-minute walk test (6MWT) [58], the Amputee Mobility Predictor with prosthesis (AMPpro) [59], and the Comprehensive High-Level Activity Mobility Predictor (CHAMP) [60]. By capturing the functional measures in each group, the effects of the ankle-foot device can be isolated from the rehabilitation effects on physical performance. These measures are as follows:

- 6MWT: the 6MWT measures the distance an individual can walk in 6 minutes without help or encouragement. It is a valid and reliable measure that correlates with physical function and has good interrater and intrarater reliability in individuals with lower limb loss [58].
- AMPpro: the AMPpro is a 21-item instrument designed to measure prosthetic mobility in individuals with lower limb loss [59].
- CHAMP: participants who attain a score of 37 or higher on the AMPpro will undergo the CHAMP, which consists of the following tasks:
  Single limb stance: participants fold their arms across their chest and then lift their foot above a 15-cm cone or box. The test ends when the foot touches the ground again (or until 30 s) or if the arms uncross. This procedure is performed on both feet.
  Edgren Sidestep Test: participants sidestep left and then right along a 5-meter line of cones (1 meter apart). The sidestep test lasts for 10 seconds.
  T-Test: the T-Test measures forward, lateral, and backward walking (or running) and sidestepping in a T pattern.
  Illinois Agility Test: this advanced agility test requires the participant to run or walk and change direction around multiple cones. Over 60 meters, participants perform 90° and 180° turns 11 times around multiple cones.

#### Powered Ankle-Foot Prosthesis Fitting

Following all baseline data collection procedures, all participants will then be fit with the Empower. Study prosthetists at VANYHHS, WRNMMC, and JAHVH are highly experienced in fitting all commercial microprocessor knees and the Empower. The study prosthetists will bench align the powered ankle-foot device onto the participant’s existing microprocessor prosthetic knee and socket. Once the powered ankle-foot device is fitted and bench-aligned to the prosthetic knee and socket, dynamic alignment of the prosthetic knee and ankle will occur. Initially, the study prosthetist will ensure proper alignment of the microprocessor prosthetic knee with the Empower turned off. The Empower will still function (ie, articulate) with the power off but will not provide net positive plantarflexion torque. The microprocessor prosthetic knee software and prosthesis alignment will be adjusted during standing and walking tasks, as necessary, until the prosthetist and the participant are satisfied with the knee alignment. Once the microprocessor knee setup is completed, the Empower will be powered on and adjusted according to the manufacturer’s specifications. Briefly, the participants will sequentially walk at 3 different speeds (slow, self-selected, and fast) while the stiffness and power delivery of the powered ankle-foot device is tuned [33]. If further dynamic adjustments to prosthetic knee alignment are necessary, these adjustments will be made at this point.

#### Powered Ankle-Foot Device Standard of Practice

Once a stable and comfortable alignment has been established, all participants will be educated by the study prosthetist on the proper use of the Empower, which includes battery handling and charging, understanding low battery indicators, considerations while driving with the Empower (if applicable), and avoidance of exposure to rain and water. Next, the participants will ascend and descend an Americans with Disabilities Act–compliant ramp under the supervision of the study physical therapist or prosthetist. The intent of ramp walking is to trigger the power for ascent but not during descent. Each participant will be given the opportunity to practice the ramp as often as necessary to ensure safe and comfortable use. The participants will then negotiate a standard staircase with handrails under the supervision of the study physical therapist or prosthetist to ensure that they can safely negotiate stairs using the Empower. Proper technique will consist of demonstrating the correct foot placement on each step to activate powered push off during ascent. Participants unable to ascend stairs in a step-over-step pattern will be shown the correct foot placement using a step-to gait pattern. For proper stair descent, participants will be shown the correct foot placement to initiate rollover and not trigger the power. Participants will be given time to practice stair ascent and descent. After stair and ramp ambulation, participants will be asked to demonstrate safe use of the Empower in different situations, including turning, varying speeds, sudden stops, obstacle avoidance, stepping over obstacles, and different surfaces. Once the physical therapist is satisfied that the participant has demonstrated safe use and all questions have been answered, the participant will be released home with the Empower. If the physical therapist feels that the progress is unsatisfactory, the participant will not take the Empower home and will be asked to return for continued supervised use until the participant demonstrates safe use. The standard of practice to use the powered prosthesis is approximately 30 to 45 minutes after fitting and tuning.

Following the baseline visit, group A will undergo the device-specific PT program, whereas group B will not undergo any further training.

### PT Program: Group A

#### Overview

Participants in group A will complete, on average, 8 PT sessions lasting 30 to 45 minutes each. The exact PT protocol and criteria for advancement are outlined by level in the subsequent section. In brief, level 1 of the PT plan will focus on education, strengthening through therapeutic exercises, and early neuromuscular reeducation. A home exercise program (HEP) will be initiated during the initial sessions and will progress along with the program. Level 2 will include gait training on level surfaces, sit-to-stand transitions, and ramp negotiation. Level 3 will include multidirectional training for both neuromuscular reeducation and gait and the introduction of stair ascent and descent. Training will conclude with level 4 where the previous skills will be further challenged and advanced gait skills, including ambulation on ramps and uneven surfaces, will be introduced. Participants must meet the outlined criteria before progressing through each level. Participants who do not meet the specified criteria will be offered additional PT sessions. The number of additional sessions will be recorded and used to refine the PT program for future use.

#### Level 1 (Sessions 1 and 2)

Level 1 (Table 1) includes initial evaluation, patient education, gait assessment, training to ensure safe use in the community, therapeutic exercises (including introduction of the HEP), and the initiation of early neuromuscular reeducation training.

**Table 1:** Level 1 device-specific physical therapy protocol.

Protocol		Description	Criteria for advancement to level 2
**Level 1 (sessions 1 and 2)**			
	Evaluation	Assessment of prosthesis fit and gait to determine deficits	N/A^a^
	Strengthening	Strengthening of transversus abdominis and multifidus, gluteus maximus, gluteus medius, and general trunk strengthening	Able to perform HEP^b^ independently
	Stretching	Address deficits from evaluation, including iliopsoas	Within normal limits for range of motion
	Education	Explanation of function of powered ankle-foot device	Verbalizes understanding of function of powered ankle-foot device
	Gait training	Safely negotiate level surface without increased falls or stumbles	Able to ambulate 46 meters without an assistive device independently on a flat, level surface; self-reported occurrence of stumbles or falls is no greater than baseline
	Mobility training	Side stepping, backward stepping, and turns	Able to ambulate outdoors without increased falls or stumbles with or without an assistive device
	Neuromuscular reeducation	Weight shifting and control over prosthesis, intact limb mobility (toes in and toes out, heel in and heel out) to promote weight shifting, static single limb balance training on prosthesis side with upper limb support, and anterior and posterior stepping exercises with the intact limb	Independent in HEP
	HEP	Initiated with all therapeutic exercises outlined in level 1	Independent in HEP

^a^N/A: not applicable.

^b^HEP: home exercise program.

#### Level 2 (Sessions 3 and 4)

Level 2 (Table 2) will include the progression of therapeutic exercises through increased frequency, duration, and resistance.

All strengthening and stretching will be shifted to the HEP by the completion of level 2. Lumbar, abdominal, and closed kinetic chain lower extremity strengthening exercises will progress to more dynamic positions. Single limb stance progressions (neuromuscular reeducation) will include decreased upper limb support for stepping exercises and progressing to step touches with the intact limb. Upper limb support will progress from bilateral support to support provided only on the side of the prosthesis. Anterior and posterior stepping exercises will begin with the support of parallel bars while maintaining weight on the prosthesis.

**Table 2:** Level 2 device-specific physical therapy protocol.

Protocol		Description	Criteria for advancement to level 3
**Level 2 (sessions 3 and 4)**			
	Evaluation	Reassessment as needed	N/A^a^
	Strengthening	Progression to sitting, quadruped, planks, standing, and transitions	Independent in HEP^b^
	Stretching	Primarily used for cooldown at completion of each session	Independent in HEP
	Education	Description of HEP and purpose of each exercise	Verbalizes understanding of each exercise
	Gait training	Improving step length symmetry (eg, verbal cueing to increase step length on nonprosthesis side) and improving rollover on prosthesis side (eg, resistive gait training with TheraBand)	Ability to trigger power in prosthetic foot during gait >50% of steps and increased gait symmetry with verbal cueing for step length as determined through observational gait analysis
	Mobility training	Transfers (eg, stand-to-sit), and ramp negotiation	Able to maintain midline center of mass with stand-to-sit transfer
	Neuromuscular reeducation	Intact limb mobility (eg, rolling ball under intact limb) to promote weight shifting, static single limb balance training on prosthesis side with minimally necessary upper limb support and stepping exercises (eg, step touches 15-20 cm step in parallel bars and step touches to a cone in the parallel bars)	Independent in HEP
	HEP	Includes therapeutic exercises outlined in level 2	Independent in HEP

^a^N/A: not applicable.

^b^HEP: home exercise program.

#### Level 3 (Sessions 5 and 6)

At level 3 (Table 3), all strengthening will be performed exclusively in the HEP. PT will include neuromuscular reeducation progression, including multidirectional movements.

Stepping exercises will be performed with decreasing upper extremity support at a tolerance demonstrated by the participant maintaining an appropriate body position. Single limb stance activities will include perturbations, such as resistance with movements of a non–weight-bearing intact limb or standing on a foam pad or balance disc. Gait training will include multidirectional stepping with upper extremity support in the parallel bars. Resistive gait training will be introduced to promote proper mechanics for loading the prosthesis in stance and achieving maximal energy return at push off. In addition, manual proprioceptive neuromuscular facilitation will be performed to promote proper anterior pelvic rotation. Multidirectional ambulation will be progressed to outside of parallel bars.

**Table 3:** Level 3 device-specific physical therapy protocol.

Protocol		Description	Criteria for advancement
**Level 3 (sessions 5 and 6)**			
	Evaluation	Reassessment as needed	N/A^a^
	Strengthening	Review as needed	Independent in HEP^b^
	Stretching	To be used for cooldown at session completion	Independent in HEP
	Education	Gait training and purpose for improving symmetry	Verbalizes understanding of gait training
	Gait training	Promoting gait initiation on prosthesis side with anterior pelvic rotation (ie, manual techniques to facilitate or initiate anterior pelvic rotation while in parallel bars with progression to anterior stepping on prosthesis side), relaxed upright posture with ambulation (ie, verbal cueing to keep chest upright), and resistive gait training with TheraBand and verbal cues for increased step length with intact limb	Demonstrates ability to trigger power with at least 80% accuracy during level ground ambulation and increased gait symmetry, including upright posture, step length, and toe break as determined through observational gait analysis
	Mobility training	Ramp and stair negotiation	Hill Assessment Index score ≥6 (ie, step past more than half foot length, with assistive device)
	Neuromuscular reeducation	Four-directional resistance exercise on intact side while maintaining single limb stance on prosthesis side, static single limb balance training on prosthesis side to be progressed to noncompliant surface (eg, foam), and progression of stepping exercises to increase time in single limb stand on the prosthesis	Able to maintain single limb stance on prosthesis side with or without an assistive device for 15 seconds
	HEP	Includes therapeutic exercises in level 3	Independent in HEP

^a^N/A: not applicable.

^b^HEP: home exercise program.

#### Level 4 (Sessions 7 and 8)

At level 4 (Table 4), PT will include advanced neuromuscular reeducation and gait training, followed by a final PT evaluation.

Neuromuscular reeducation will include single limb squats in the parallel bars with upper extremity support, as needed. Gait training will continue resistive training on even surfaces, proprioceptive neuromuscular facilitation for pelvic rotation, verbal and tactile cueing for symmetrical and appropriate trunk rotation, and negotiation of uneven surfaces.

Individuals who do not meet the criteria to complete the PT program will be offered an additional 8 PT sessions after completion of the study.

**Table 4:** Level 4 device-specific physical therapy protocol.

Protocol		Description	Criteria for advancement
**Level 4 (sessions 7 and 8)**			
	Evaluation	Re-evaluation at final session	N/A^a^
	Strengthening	N/A	N/A
	Stretching	To be used for cooldown at session completion	Independent in HEP^b^
	Education	Importance of continuation of HEP	Verbalizes understanding
	Gait training	Promoting gait initiation on prosthesis side with anterior pelvic rotation, manual techniques and verbal cues to promote increased trunk rotation and trunk rotation symmetry, verbal cueing for symmetrical arm swing and trunk rotation (eg, “relax your shoulders”), and resistive gait training with TheraBand and verbal cues for increased step length with the intact limb and relaxed upright posture	Demonstrates ability to trigger power with at least 90% accuracy for powered prosthetic foot and increased gait symmetry for trunk rotation and arm swing, as determined through observational gait analysis
	Mobility training	Ascending and descending ramps and stairs	Stair Assessment Index score of at least 4 (with assistive device, step-to pattern) for ascending and descending stairs, and ambulates in the community without increased participant-reported stumbles or falls compared with baseline
	Neuromuscular reeducation	Applied during HEP	Demonstrates increased gait symmetry for step length, push off, anterior pelvic rotation, upright posture, trunk rotation, and arm swing
	HEP	Review HEP	Demonstrates independence in HEP

^a^N/A: not applicable.

^b^HEP: home exercise program.

### Data Collection Visit 2

After completion of the device-specific PT program, all participants will undergo data collection on the powered ankle-foot prosthesis. The participants will repeat the quality-of-life assessment, subjective surveys, pain assessment, cognitive load assessment, biomechanical gait analysis, and functional measures using the powered ankle-foot prosthesis, as described in the baseline visit. Following visit 2, participants will keep the powered ankle-foot prosthesis for an additional 4 weeks of home use and community use but will not undergo any further device-specific training.

### Data Collection Visit 3

After the final 4 weeks of powered ankle-foot prosthesis use, participants will undergo final data collection. Participants will repeat the quality-of-life assessment, subjective surveys, pain assessment, cognitive load measurements, neurocognitive assessment, biomechanical gait analysis, and functional measures, as described in the baseline visit. Following data collection, all participants will be refitted with their energy storing and returning ankle-foot devices, and the powered ankle-foot devices will be returned to the study staff.

### Statistical Analysis

Across the study population, outcomes will be assessed with descriptive statistics and compared between each ankle-foot device category as well as by PT intervention (ie, device-specific PT and standard of care). Inferential statistics for ordinal data will be conducted with a repeated-measures Friedman test (α=.05) and a Dunn post hoc test at a 95% CI. To address which measures are the most sensitive to intervention type, a linear mixed-effects model will be used. Separate models will be used for each type of measure (pain, subjective, cognitive, neurocognitive, functional, and biomechanical), and measures that have a significant association with the intervention type in the presence of adjusting (control) variables will be determined. Pair-wise comparisons will be tested for significance using linear contrasts with a Tukey honestly significant difference or by applying a Bonferroni correction, where applicable. The following sections outline the specific analyses that will be performed for each study objective.

### Planned Statistical Analysis for Biomechanical and Functional Outcomes

Although there are numerous biomechanical and physiological parameters that can be evaluated following gait analysis [61], this investigation will focus on the biomechanical parameters that are most relevant, commonly used, able to discriminate, and have specific clinical relevance for individuals with transfemoral limb loss. The primary biomechanical outcome measures will include measures of rollover shape, individual characteristics of the 3D ground reaction force, and ankle, knee, and hip joint angles, moments, and powers (on both the intact and affected limbs). To evaluate the load distribution of the medial and lateral knee compartments, the peak resultant ground reaction force, ground reaction force rate, peak knee external adduction moments, and knee external adduction moment rate will be compared between the baseline (passive energy storing and returning condition) and the powered condition at each follow-up visit.

Linear mixed-effects models will be used to identify statistically significant differences in gait temporal-spatial and biomechanical variables for all walking speeds. The fixed effects will be the average differences in gait biomechanical and temporal-spatial variables by prosthetic ankle type (powered vs passive). These models also estimate random effects because of differences in mean biomechanical variables across participants, as well as the random effects associated with minimized variability, as the participants will be tested with both prostheses. For example, a linear mixed-effects regression will be used to examine the relationship between the intact knee peak external adduction moment and the prosthetic ankle-foot condition. Peak intact knee external adduction moment will be the dependent variable, whereas ankle-foot condition will be the independent variable, and participant-by-ankle-foot condition will be modeled as random effects. Pair-wise comparisons will be tested for significance using linear contrasts with a Tukey honestly significant difference or by applying a Bonferroni correction, where applicable. In addition, linear mixed-effects regression will be used to determine the association between prosthetic peak ankle power and foot condition.

### Planned Statistical Analysis for Pain Outcomes

The following parameters will be measured and statistically compared between baseline (energy storing and returning condition) and each follow-up visit with the powered ankle-foot prosthesis (weeks 4 and 8):

- Joint reaction forces on the lower back (L5 and S1) and contralateral (intact) knees
- PEQ pain scores
- Functional outcome measures (6MWT, AMPpro, and CHAMP)

Spearman correlations will be calculated to correlate data between pain and lower extremity kinematic and kinetic parameters of interest and pain and functional outcome values across the groups. Linear mixed-effects models will be used to identify statistically significant differences in pain scores and biomechanical variables listed in the previous section for all walking speeds. For example, a linear mixed-effects regression will be used to examine the relationship between peak reaction moments at L5 and S1 and pain for each prosthetic ankle-foot condition. Pair-wise comparisons will be tested for significance using linear contrasts with a Tukey honestly significant difference or by applying a Bonferroni correction, where applicable.

Multiple linear regression will be performed with PEQ pain as the dependent variable. Because of the large number of predictor variables in relation to the number of participants, penalized methods (eg, ridge regression or lasso) will be used to identify variables that contribute the most to the prediction model. Penalized methods add a tuning parameter to the regression model that shrinks the less important coefficients toward 0. Cross-validation will be used to select the best tuning parameter value [62]. The independent variables will be comprised of functional parameters (eg, peak joint reaction forces) and the condition (ankle-foot type and intervention).

### Planned Statistical Analysis for Cognitive Load and Neurocognitive Outcomes

Linear mixed-effects models will be performed to examine the differences between the ankle-foot devices on cognitive performance, walking speed, and subjective responses to attention. The parameters in the linear effects model will include prosthetic ankle-foot type, PT intervention, and participants. Ankle-foot type, PT intervention, and cognitive performance will be treated as fixed effects, whereas participants will be treated as random. The total number of errors and the error rates for the cognitive task will be calculated, and the mean error rate will be determined for each cognitive task performed by each participant. Repeated-measures ANOVA will be used to compare the error rates for the 3 cognitive tasks. Fisher least significant difference test will be used to make post hoc comparisons.

For the neurocognitive battery, Pearson correlation coefficients (pair-wise 2-tailed) will be calculated for all variables of interest. Stepwise multiple regression will be performed with the neurocognitive scores (Neurocognitive Index, composite memory, cognitive flexibility, and complex attention scores) as the dependent variable. The independent variables will comprise functional outcomes, pain, and gait biomechanics, including asymmetry index.

### Power Analysis and Sample Size Estimation

The sample size was based on a power analysis of 3 biomechanical measures (leading limb work, ground reaction force rate, and knee adduction moment rate) and 1 functional outcome (6MWT distance), and 1 subjective outcome (PEQ—utility) obtained from preliminary analyses, with all measurements obtained at baseline and 2 additional measurements over an 8-week period. The sample size was calculated for the group-by-time interaction, which tests the differences in change over time between the study groups. Assuming an α error rate of 5%, Table 5 presents the power achieved for each measurement for 30 participants, 15 (50%) in each group.

**Table 5:** Power analysis and sample size estimation.

Measure	Difference, mean (SD)	Power (N=30)
Leading limb work (J/kg)	0.03 (0.04)	85
Ground reaction force rate (N/kg/s)	15 (26.9)	82
Knee adduction moment rate (N m/kg/s)	1.1 (1.2)	98
6MWT^a^ distance (m)	60 (61)	96
PEQ^b^—utility	8.4 (8.8)	96

^a^6MWT: 6-minute walk test.

^b^PEQ: Prosthesis Evaluation Questionnaire.

## Results

This study was funded in September 2017, with enrollment beginning in September 2018. Data collection is expected to conclude by March 2024. Data analysis of the completed data set is expected to begin after final data collection. The initial dissemination of results is expected in August 2024, with subsequent publication of secondary analyses in December 2024.

## Discussion

### Expected Outcomes and Anticipated Principal Findings

After the completion of this research project, this investigation will have quantified the dependence of symmetrical lower limb gait biomechanics, physical function, and pain reduction on advanced prosthetic technology and device-specific rehabilitation. Furthermore, a device-specific treatment strategy designed to minimize impairments and maximize function will be evaluated. Finally, an objective measure of cognitive load and neurocognition to guide the prosthetic prescription of powered ankle-foot prostheses will be assessed. As such, the evidence-based outcomes obtained from this research investigation can be appropriately translated into clinical practice as well as drive the future of clinical care in this population.

VA provides care for veterans with limb loss of all generations, including the influx of service members with limb loss from the most recent conflicts [5]. Projected trends indicate that the overall number of individuals with limb loss will continue to increase dramatically, largely attributable to the aging population and the growing number of people with dysvascular disease and diabetes [1]. With this large population expected to grow, considerable resources will be required for rehabilitation and prosthetic services, driving limb loss care to become a high priority for VA. Effective outcomes-based clinical practice will be necessary to decrease long-term disabilities associated with prosthetic use and improve the quality of life. Therefore, it is the goal of this study to examine the effectiveness of a powered ankle-foot prosthesis and device-specific rehabilitation on gait biomechanics, performance, and pain in individuals with transfemoral limb loss. Results from this investigation will provide evidence-based outcomes that can be translated into successful strategies to minimize impairments and maximize function and may drive the evaluation of future advancements in prosthetic technology.

### Dissemination Plan

The results of this investigation can help form evidence-based guidelines for individuals with transfemoral limb loss that can serve as a source for lower limb loss clinical practice guidelines. Dissemination of the results of this study within the DoD, VA, and the civilian health care systems will be performed in 3 ways. First, the results will be disseminated to the scientific and clinical community through traditional means, such as peer-reviewed submissions to professional conferences (eg, the Gait and Clinical Movement Analysis Society Annual Conference, the American Congress of Rehabilitation Medicine Annual Conference, and the Military Health System Research Symposium), targeted limb loss and rehabilitation publications (eg, *Archives of Physical Medicine and Rehabilitation*, *Gait & Posture*, and *Frontiers in Bioengineering and Biotechnology*), as well as sharing the data through large data repositories. Second, both VA and the DoD have national, interdisciplinary groups and committees that enable the national dissemination and adoption of best practices among different disciplines. For the VA and DoD limb loss care teams, the Extremity Trauma and Amputation Center of Excellence and the VA Amputation System of Care hold a bimonthly webinar series for clinicians, scientists, and researchers that is available across the entire DoD and VA health care network. This series allows research results to be presented to a large, diverse audience of researchers and health care professionals in limb loss care, which can directly influence the care provided to veterans and service members with limb loss. Finally, the outcomes will be disseminated directly to leaders in the prosthetics industry to provide real-world feedback on their products. The results provided to industry leaders can help in the evolution of lower extremity prosthetic components, which can then lead to improved devices for individuals with transfemoral limb loss.

### Limitations

This investigation will not address the varied dosing, timing, frequency, and duration of the device-specific rehabilitation protocol. However, the frequency of PT visits and protocol timing will be evaluated for each participant, which can provide preliminary data for a future study to optimize the rehabilitation strategy. The heterogeneity of the sample population may also limit the generalizability of the outcomes to a more diverse population. In addition, the statistical analysis models will also adjust for specific parameters (eg, age, time since limb loss, and etiology of limb loss), which may limit the sample size and interpretability of the results. The type of microprocessor knee used by each participant will not be prescribed and may be a confounding factor. This influence of the microprocessor knee type will be evaluated in the statistical models. Finally, a 4-week assessment following completion of the device-specific protocol may not be sufficient to evaluate any PT rebound effects or long-term changes in gait, specifically regarding the effectiveness of the acute PT intervention on gait biomechanics.

## Appendix

Multimedia Appendix 1 Peer-review summary statement.

Multimedia Appendix 2 Peer-review response from the investigators.

## Abbreviations

6MWT: 6-minute walk test
AMPpro: Amputee Mobility Predictor with prosthesis
CHAMP: Comprehensive High-Level Activity Mobility Predictor
DoD: Department of Defense
HEP: home exercise program
IRB: institutional review board
JAHVH: James A. Haley Veterans’ Hospital
PEQ: Prosthesis Evaluation Questionnaire
PT: physical therapy
VA: Veterans Affairs
VANYHHS: Veterans Affairs New York Harbor Healthcare System
WRNMMC: Walter Reed National Military Medical Center

## Disclaimer

The views expressed in this paper are those of the authors and do not necessarily reflect the official policy of the departments of the Air Force, Army, Navy, Defense, Veterans Affairs, or the United States government. The identification of specific products or scientific instrumentation is considered an integral part of the scientific endeavor and does not constitute endorsement or implied endorsement on the part of the author, the Department of Veterans Affairs, the Department of Defense, or any component agency.

## Data Availability

All data for this manuscript are included in this published paper. Final results will be available via a publicly available data repository.
