# Supplementary material for: Effects of a Powered Ankle-Foot Prosthesis and Physical Therapy on Function for Individuals With Transfemoral Limb Loss: Rationale, Design, and Protocol for a Multisite Clinical Trial
Source: JMIR Res Protoc. 2024 Jan 26;13:e53412. doi: 10.2196/53412 (PMC10858430; doi:10.2196/53412)
Supplement: Multimedia Appendix 1 [file resprot_v13i1e53412_app1.pdf]

Department of Defense
U.S. Army Medical Research and Materiel Command
Congressionally Directed Medical Research Programs
Fiscal Year 2016 Orthotics and Prosthetics Outcomes Research Program
Prosthetics Outcomes Research Award - Funding Level 3 - Clinical Trial
Peer Review Summary Statement

CDMRP Log Number: OP160073
Grants.gov ID Number: GRANT12289883
Meeting Dates: 01/22/2017-01/23/2017
Review Panel: Prosthetics Outcomes Research - 2

Project Duration: 36 months
Total Budget Requested: $1,994,596
Direct Costs: $1,735,941
Indirect Costs: $258,655

Title: Effects of a Powered Ankle-Foot Prosthesis and Device-Specific Physical Therapy on Function and Pain for Individuals Living With Transfemoral Limb Loss
Principal Investigator: Jason Maikos
Performing Organization: Narrows Institute for Biomedical Research
Contracting Organization: Narrows Institute for Biomedical Research

Overview

The Principal Investigator (PI) of this application proposes to determine if powered plantar flexion coupled with a device-specific physical therapy (PT) intervention will result in improved biomechanical gait kinematics and kinetics and will correlate with a decrease in pain and improved functional performance in individuals with a transfemoral amputation (TFA). An 8-week randomized clinical trial will be performed. The intervention is an 8-session intensive device-specific PT intervention in addition to standard care. The projected outcomes are gait symmetry, functional efficacy, pain, and neurocognitive functioning. The project's specific aims are to (1) examine the effect of a device-specific PT intervention on kinematic and kinetic patterns and functional efficacy of powered ankle-foot prostheses for individuals with TFA compared to the current standard of practice; (2) correlate the contribution of a powered prosthetic ankle-foot device and device-specific PT with changes in pain; and (3) determine if neurocognitive function is a limiting factor in improvement in functional outcomes, gait symmetry, and pain achieved through powered prosthetic devices and/or PT.

	**Average Score**	**Standard Deviation**
**Overall Evaluation**<br>Rating Scale: 1.0 (highest merit) to 5.0 (lowest merit)	1.9<br>**(Excellent)**	0.1
**Evaluation Criteria**<br>Rating Scale: 10.0 (highest merit) to 1.0 (lowest merit)	**Average Score**	
**Research Strategy and Feasibility**	6.7	
**Military Benefit and Impact**	7.8	
**Personnel**	7.6	
**Transition Plan**	7.1	

Procurement Sensitive Document
Do not copy or distribute without CDMRP written permission.
Prepared by SRA International, Inc., a support contractor to CDMRP

SCORED EVALUATION CRITERIA

Research Strategy and Feasibility
Average Score: 6.7

Scientist Reviewer A

The study will use an 8-week multicenter design with stratified random sampling to create 2 groups (n = 15 per group) of individuals with TFA. Group A will be provided with a powered prosthetic ankle-foot device with an 8-session intensive device-specific PT intervention; group B will be provided with a powered prosthetic ankle-foot device without the device-specific PT. A full gait analysis, pain assessment, and functional assessment will be completed at baseline (using the participant's current passive prosthesis) and again at 4 and 8 weeks of powered device use. Cognitive and neurocognitive testing will be conducted for all participants at baseline and at study completion.

Strengths: The proposed research strategy has numerous major strengths. There is ample support for this study and use of a power ankle for individuals with TFA; a sound evidence-based rationale is provided. The study is especially relevant to addressing the Orthotics and Prosthetics Outcomes Research Program focus area "Limited current technologies…for the rehabilitation…of function that optimize patient interaction, usability…." Preliminary data with the powered ankle-foot prosthesis (BiONX BiOM) by this PI from an ongoing longitudinal study (12 months) with transtibial amputation is near completion; the knowledge gained from that work and a similar case study conducted in an individual with TFA strengthens this application. The recruitment strategy allows for a heterogeneous sample—males and females with trauma, dysvascular, and/or congenital cause of limb loss. The potential to have significantly different groups for intervention should be controlled for with the proposed means of stratified sampling. Inclusion and exclusion criteria are soundly constructed, and there is a full description for handling confounds such as medication use and other interventions during the time of study. There is control for standardizing prosthetic fitting and adjustment throughout the study. The described methods include sufficient safeguards during all aspects of the study, including the intervention program, and the documentation addresses all ethical and regulatory standards of human research. There are sufficient plans for protection of data sharing/management. The statistical plan comprehensively outlines the measures to address all hypotheses (and subhypotheses) for comparisons between the 2 groups. Power calculations are described and consider clinically meaningful change for selected dependent variables. All functional tests are reliable and valid for the proposed population of study. The selected spinal innervation area of L5/S1 (the low back) for comparing gait and pain is well thought out (it is the most frequently reported area of low back pain in this population). Walking speeds are effectively controlled for. Baseline measures and completion-of-study measures with currently used nonpowered ankle/foot will serve as reference for change and may allow for meaningful clinical differences to be determined. There is control for potential ceiling effects for the functional measure (use of the Comprehensive High-Level Activity Mobility Predictor [CHAMP] instead of the Amputee Mobility Predictor [AMP]). The neurocognitive tests implemented during walking are novel for this population, as is the use of a baseline and posttest neurocognitive battery index assessment for additional exploration. There is sound selection of auditory and verbal tests only (avoidance of reading tasks) to ensure safety during walking. There is a strong distinction between group interventions with no apparent risk for cross-contamination of training techniques for the power ankle, which is distinctly different from the standard care plus the PT intervention program. The PT program is comprehensively designed and includes characteristics of current standards of PT care for individuals with TFA; the intervention also addresses different capabilities of a power ankle versus a standard energy-storing foot. There are appropriate criteria that should ensure standardization for the individualized progression of exercise, and there is an organized structure plan for advancement of exercise each week of the 4-week program.

Weaknesses: There are a few minor weaknesses noted. There is no consensus (supportive evidence) on the best PT practice in this area—the intervention is extrapolated from previous research/experience. The preference and/or weight of the power ankle for the population of the study may influence results—especially with the dysvascular population. A power analysis to determine sufficient sample size for pain and neurocognitive testing is not provided.

Scientist Reviewer B

Strengths: The research strategy is clearly outlined and logically organized. Overall, the background is well written, displays a sound understanding of the topic area, and provides a firm foundation for this study. In objective 1, the application clearly states the intent to determine the effects of a powered prosthetic ankle-foot device and PT intervention on lower- limb mechanics, functional efficacy, and pain for persons with TFA. In objective 2, the applicants also intend to develop preliminary rehabilitation guidelines to aid this patient population and aid in better prescription for powered ankle-foot devices. The first objective is clearly supported by the 3 specific aims and specific hypotheses that are proposed to investigate each aim. These specific aims are independent and well supported by appropriate methodology, variables, and statistical plan. Appropriate preliminary data for lower-limb kinematics and kinetics and functional tests are presented, increasing the feasibility for Aim 1 of the study. The PT aspect of Aim 1 is supported through the inclusion of a detailed device-specific PT plan. Aims 2 and 3 are not as well supported, but the methods appear appropriate and are supported through literature citations. For example, Aim 2 will attempt to quantify changes in pain and will use the Patient-Reported Outcomes Measurement Information System (PROMIS) Pain Scale in addition to the traditional visual analog scale. Recruitment can be difficult within this patient population, but the applicants make a convincing case through citation of ongoing investigations and the track record of their 2 institutions that they will be able to recruit the number of patients needed. In the event they are unable to recruit the numbers they need, they provide an alternative plan for opening up their recruitment and budget for transport for patients further from their institutions. The recruitment process is well outlined, and the applicants appear to have adequate access to their population and an appropriate plan in place for recruiting participants. The application appropriately addresses risk mitigation and the informed consent process. The application also has an adequate plan in place for securing patient data.

Weaknesses: There are a number of moderate weaknesses with the study design. While objective 1 is well supported through the 3 aims, objective 2, to develop preliminary rehabilitation and selection guidelines, is not well supported. The information collected as part of Aims 1 through 3 will be useful to develop guidelines, but no information is provided to detail how the data will be translated into guidelines. Another weakness comes from the small sample size (30 participants) and the limited information used to support this value from the power analysis. Aim 1 calls for 2 different groups to test the effect of the device-specific PT, so this leaves groups of only 15 participants. Some support is provided to suggest this is a large enough sample for the gait mechanics aspect of the project but not for the pain and cognitive testing that will also take place. Additionally, it is not clear why the study is only seeking 30 participants over 3 years when the application states that 150 individuals with amputations have been treated at the Walter Reed National Military Medical Center (WRNMMC) through the first quarter of 2016. The length of follow-up is also not well supported. The protocol includes pilot data to suggest normalization of lower-limb biomechanics after 180 days of powered ankle usage in persons with transtibial amputations. However, the proposed study will test patients at baseline, after 4 weeks, and after 8 weeks. There is a good chance that patients are still adjusting to their new limb and that further normalization between the 2 patient groups would continue past the 8-week mark. With a 3-year study and full support for a physical therapist and research engineer and half support for a research coordinator, there is no reason longer-term data cannot be collected on this cohort of 30 patients. Also, a true baseline of the patients when they first receive their powered limb will not be taken. There will be no data to help

determine who was a quicker adapter to the new limb. The division of work between the 2 institutes is not well described. It is unclear if the device-specific rehabilitation will be instructed by 1 person at a single institution or different people at the 2 institutions. This would add another level of variance to the data and again work against the small sample size. Additional concerns exist about the large weight of the foot, and this might be difficult for older and/or weaker patients.

Biostatistician Reviewer

Strengths: The study design is clearly described. Many outcomes addressing the specific aims will be measured. The participants will be followed for 8 weeks with measurements made every 2 weeks. Mixed-effects ANOVA and mixed-effects linear models will be used. The group by time interaction term will be of interest. Multiple linear regression will be performed with a pain visual analog scale as the dependent variable. Because of the large number of predictor variables in relation to the number of participants, penalized methods (eg, ridge regression, lasso) will be used to identify variables that contribute the most to the prediction model. Penalized methods add a tuning parameter to the regression model that shrinks less important coefficients toward zero. Pairwise comparisons will be tested for significance using linear contrasts with Tukey's honestly significant difference or applying Bonferroni correction. The statistical methods are appropriate and correctly applied. The applicants will use corrections for multiple comparisons. Diligence is expressed with regard to data security and compliance with the Health Insurance Portability and Accountability Act (HIPAA).

Weaknesses: The sample size calculation cannot be checked without further information, not given, such as the baseline variance and autocorrelation.

Military Benefit and Impact
Average Score: 7.8

Scientist Reviewer A

Strengths: The military benefit and impact have numerous major strengths, and the study is novel in that it appears to be the first study involving device-specific PT intervention and applicability of using the BiONX BiOM in individuals with TFA. The sample for the study will be recruited from 2 major medical centers; these sites offer excellent potential for the inclusion of participants that are directly representative of current service members and veterans (and the general civilian population). The potential to determine the establishment of a device-specific PT program is significant in that the program may allow for shortened recovery periods and improved outcomes for injured service members and veterans. This very innovative project is directly designed to build upon the earlier work of this team to examine the benefits of utilizing advanced technologies in service members and veterans with limb loss. The results can be immediately implemented into the clinic within the Department of Defense and Veterans Administration (VA) health care systems, and the results of neurocognitive testing may further assist in developing preliminary guidelines for prescription of powered prosthetic feet/ankles. If the intervention is effective, the long-term outcome of improved walking patterns may in turn reduce the risk of acquiring secondary musculoskeletal health outcomes, such as low back pain or arthritis, and thus impact the quality of life for servicemen and veterans—as well as the general population.

Weaknesses: There are a few minor weaknesses noted in this area of impact, including the limited generalizability of findings and that the long-term impact cannot be readily ascertained. The cost for use of the powered prosthetic ankle-foot device is a minor weakness, but there are many potential benefits that may outweigh the cost.

Procurement Sensitive Document
Do not copy or distribute without CDMRP written permission.

Scientist Reviewer B

Strengths: The objectives of this study are to learn more about the biomechanical and cognitive impacts of wearing a powered ankle and receiving device-specific PT so that recommendations can be made toward prosthetic selection and rehab. The need for this study is well supported through discussion of the increasing number of amputations that are occurring as a result of war and diabetes. Additionally, nice support is given for the impact of gait asymmetry and limb overload on long-term health effects such as osteoarthritis. As a result of the proposed research, more information about the impacts of device-specific therapy will be available to further improve patient care and refine the criteria used to fit patients with the proper prosthetic device. The applicants do a nice job of placing their proposed study in the context of recent research calls from the Department of Defense. Overall, the applicants make a convincing case for the benefits of their proposed research.

Weaknesses: The limited number of participants will make it difficult to extend the data obtained in this study to amputees with different demographics from those used in this study. Also, the impacts on long-term outcomes will not be investigated with this study, limiting the impact of the research. Additionally, no specific numbers are given as to the total patient population who have a TFA and who would likely benefit from a powered ankle. The size of this cohort is unclear, as are the potential financial benefits that might occur as a result of this study. These concerns are fairly minor.

Consumer Reviewer

Strengths: If successful, this study is going to show whether PT benefits persons with TFA that use powered foot/ankle prosthetic devices more than those with TFA that do not receive PT. The application adequately describes the potential immediate and long-term effects on patient care by hypothesizing that powered foot/ankle prosthetic devices, coupled with skilled PT, is the best standard of care for TFA. The potential benefits, both short and long term, promise positive clinical outcomes with maximum usability of powered prosthetic devices among TFA. Subsequently, affected populations, their family members, and local communities can expect higher mobility and qualities of life socially, professionally, and socioeconomically. The current standard of care is to prescribe new prosthetic feet upon request, but PT is not prescribed as prosthetic componentry changes or gets updated. This study could change prosthetic care to ensure users receive skilled PT as a requirement of receiving a motorized powered ankle. It is dangerous to patients to prescribe the powered ankle and ignore skilled PT. TFAs require new uses of existing musculature to safely and efficiently use this powered ankle.

Weaknesses: The weakness is outfitting group B (15 users) with a complex powered prosthetic device and zero PT. The protocol promises user instructions, but a powered foot connected to a prosthetic knee is a potentially dangerous mechanical setup for a “basically trained” user, whose muscles are comfortable (complacent) with passive devices, relying solely upon the user prompts. Motorized feet/ankles, on the other hand, react to various stimuli, often spontaneously compared to the passive devices, causing unpredictable instability when connected to passive prosthetic knees (both microprocessor and mechanical) vice motorized knees. Motorized knees are programmed to react appropriately to the connection of powered ankles; however, this study does not include motorized knees.

Personnel
Average Score: 7.6

Procurement Sensitive Document
Do not copy or distribute without CDMRP written permission.

Scientist Reviewer A

The PI, Jason Maikos, received a doctoral degree in biomedical engineering from Rutgers State University in 2007 and a bachelor of science degree in chemical biology in 2001 from Stevens Institute of Technology. He completed a postdoctoral fellowship for the New Jersey Commission on Science and Technology in 2010. Dr Maikos is currently the director of the VISN3 Gait and Motion Analysis Laboratory at the Veterans Affairs New York Harbor Healthcare System (VA NYHHS).

Strengths: During the PI's postdoctoral studies he was working on innovative technology, and he has conducted other studies with a number of the collaborative team members for this study. The PI is currently studying short- and long-term effects of utilizing a powered ankle-foot prosthesis for individuals living with transtibial amputation; therefore, he has prior experience with the current advanced technology. This collaborative team is multidisciplinary and well established. The key personnel have recent experience, funded studies, and publications in research related to the proposed study; most have collaborated with the PI in other investigations. The physical therapist/prosthetist has over 15 years of clinical practice experience as well as experience in the fit/function of the BiONX power ankle; he has published research with the power ankle. The physical therapist has over 9 years of experience working with service members and veterans with limb loss and will effectively be able to implement and oversee a PT assistant in implementing the outlined PT intervention program. The level of effort appears to be appropriate for each member of the team, including bioengineers for the gait laboratory and biostatistician for successful implementation of this study.

Weaknesses: Although this PI is actively engaged in research with a number of publications and is working on other grants of similar design, he is not yet well established in dissemination of this work through publication as primary author. This is a minor weakness.

Scientist Reviewer B

Strengths: The research team comprises individuals with biomedical engineering, rehabilitation, kinesiology, PT, and prosthetics backgrounds that will help them successfully tackle this project. Additionally, the team has budgeted for a biostatistician to ensure the protocol is properly developed and the results properly analyzed. Dr Maikos, the proposal PI, is the director of the VISN3 Gait and Motion Laboratory and has a very extensive track record of successfully completing similar projects and presenting the results from these projects in journals and conferences. Dr Maikos has assembled an impressive team of engineers and clinicians that includes Dr Christopher Dearth, research director at WRNMMC and facility research director at the Department of Defense–VA Extremity Trauma and Amputation Center of Excellence (EACE), and Dr Bradford Hendershot, laboratory director and assistant professor at the Uniformed Services University of the Health Sciences. These members, as well as others, including a very accomplished PhD candidate (Alison Linberg), make for a very prepared and accomplished group. This team has great resources at their disposal and the experience to use it.

Weaknesses: This team along with the additional personnel of research coordinators and other supporting members is possibly too large for the project being proposed. Only 30 participants are being recruited, and only 15 of those participants will receive the in-depth device-specific therapy. The personnel of this study includes a full-time research engineer for 3 years, a research physical therapist for 6 months a year, and a research coordinator for 6 months a year at the New York Harbor Healthcare System. Additionally, full support for 3 years for a physical therapist and 75% for a biomedical engineer for 3 years are requested. This is a great research team, but the percent efforts for these positions are too large for the limited-size study being proposed. For example, the research coordinator will only have to recruit less than 2 participants a month to fulfill the enrollment of this study. The physical therapist and PT assistant

only have to provide instruction for the device for 15 people and basic instruction for an additional 15 people over the course of 3 years.

Biostatistician Reviewer

Strengths: The PI, Jason Maikos, and his Coinvestigators are experienced, trained, funded, and accomplished in topics relevant to this application. This team has demonstrated their ability to communicate with each other and collaborate, as evidenced by coauthored manuscripts listed in PubMed. Dr Maikos and Dr Nelson recently published a meta-analysis on outcomes associated with the intrepid dynamic exoskeletal orthosis, for example. These investigators are therefore qualified and able to conduct this study successfully.

Weaknesses: No weaknesses were noted.

Transition Plan
Average Score: 7.1

Scientist Reviewer A

Strengths: The strategy (and funding) is effective for bringing preliminary work and a case study on the power ankle in individuals with TFA to the next level. This study has strong potential to create a long-term benefit as a major source of evidence-based clinical practice for fitting individuals with TFA with a powered ankle-foot system and for guiding future device-specific gait training. In addition, there is a strong potential for future research to determine a long-term relationship between improved gait with a power ankle and reduction in secondary musculoskeletal health conditions such as low back pain and knee and hip osteoarthritis. The investigators have well-established collaborative partnerships for potential future research, and there are outlined plans to facilitate the immediate implementation of results into VA, Department of Defense clinics, and EACE and also by establishing a national training program for rehabilitation providers at the Regional Amputation Center sites across the VA system. There are outlined plans for dissemination of finding in research presentations and publications. The schedule and milestones for follow-up research are realistic and feasible.

Weaknesses: There are a few minor weaknesses that affect the potential for long-term transition. The application does not include information regarding potential risk analyses for cost and sustainability of the study except for sharing results of the second generation of the BiOM, now named emPower, for continuity and sustainability of using power ankle units. Even if findings of this study are positive, the cost of powered ankle systems is great and might not be readily be available for individuals in the military or civilian sector.

Scientist Reviewer B

Strengths: The transition plan provides information for how the research results will be made available to the military community, the medical device industry, and to the prosthetics community at large. The applicants provide excellent detail for how they can leverage their connections with EACE and Amputation System of Care to disseminate the research to active military and veterans. Great specifics are provided regarding monthly calls and webinar series that take place between primary clinicians and members of the military prosthetics community. Based on these specifics and the unique connections of the members involved with this study, there is little doubt that the results can be effectively communicated to the military community. The applicants also make a convincing case that these results will be of value to the medical device companies producing powered prostheses. The applicants also

Procurement Sensitive Document
Do not copy or distribute without CDMRP written permission.

detail appropriate journals and conferences where this information could be passed to the prosthetics community at large.

Weaknesses: The transition plan would be stronger if more specifics were given as to the roles each research team member will play in the dissemination of this research. Additionally, more details could have been provided in the transition plan for communicating with the general prosthetics and industry communities.

Technology Transfer Specialist Reviewer

Strengths: There is no technology transfer involved in this study, and no US Food and Drug Administration (FDA) IDE regulatory oversight is needed. As a result, there should not be any barriers or time necessary for technology transfer and FDA regulatory activities. Milestones and schedules for this study are part of the clinical aspects of this proposal.

Weaknesses: A discussion is needed about how the ankle-foot prosthetic would be funded, which is important in terms of continued research. Also, there is some concern about dissemination strategies and communication with industry.

UNSCORED EVALUATION CRITERIA

Environment

Scientist Reviewer A

The scientific environment at the VA NYHHS and WRNMMC is fully supportive of this research proposal, and the PI has sufficient access to all of the needed resources. Both sites have prosthetics and orthotics services that are fully staffed and have equipped laboratories for the design, fabrication, and fitting of advanced prosthetic technology, including the BiONX power-ankle. There is excellent support of PT services in both facilities; the space and equipment resources are more than sufficient to implement the device-specific PT training program. Both facilities have gait and motion analysis laboratories that offer state-of-the-art data collection.

Scientist Reviewer B

The facilities at the collaborating locations appear to be more than adequate to allow for the successful completion of this project. There is evidence of appropriate institutional support. Overall, the resources, infrastructure, and collaborations appear to be a strength of this proposal.

Budget

Scientist Reviewer A

The proposed budget for resources, personnel, etc, and duration is appropriate for completion of this study. Subaward costs for collaborators at The Henry Jackson Foundation for the Advancement of Military Medicine, Inc, are included and appropriate for support.

Scientist Reviewer B

The budget for personnel is excessive.

Application Presentation

Scientist Reviewer A

Overall, the application is complete, organized, and well written. All tables and figures enhance the quality of this application. The abstract could have been organized a bit better. Those are minor issues that did not affect the overall evaluation of the application.

Scientist Reviewer B

The application is clearly written and easy to follow. The presentation of the application did not influence the evaluation of this submission.

Procurement Sensitive Document
Do not copy or distribute without CDMRP written permission.
