# Supplementary material for: Effects of a Powered Ankle-Foot Prosthesis and Physical Therapy on Function for Individuals With Transfemoral Limb Loss: Rationale, Design, and Protocol for a Multisite Clinical Trial
Source: JMIR Res Protoc. 2024 Jan 26;13:e53412. doi: 10.2196/53412 (PMC10858430; doi:10.2196/53412)
Supplement: Multimedia Appendix 2 [file resprot_v13i1e53412_app2.pdf]

**Peer and programmatic Reviewer Concerns Response**

*1) The weakness is outfitting group B (15 users) with a complex powered prosthetic device and zero PT. The protocol promises user instructions, but a powered foot connected to a prosthetic knee is a potentially dangerous mechanical setup for a "basically trained" user, whose muscles are comfortable (complacent) with passive devices, relying solely upon the user prompts. The GOR advises including some sort of PT regimen with a human use protocol.*

**RESPONSE:** Participants who are randomly enrolled in Group B (15 participants) will receive a powered ankle-foot device and will also undergo the current standard of care for a powered ankle-foot device at the Department of Veterans Affairs. To ensure the safety of participants, first, the powered ankle-foot device has been cleared by the FDA for use in individuals with transfemoral limb loss. Any clinical patient with transfemoral limb loss who is prescribed the powered ankle-foot device at the Department of Veterans Affairs would receive the same standard of care provided in this study. In addition, participants in this study are also required to meet a minimum standard of function and must score 33 or higher on the Amputee Mobility Predictor with Prosthesis (AMPPRO). This score corresponds to a high K2 or higher level ambulator, which means that participants at this level will have higher ability for ambulation in the community. Participants will all also be experienced microprocessor knee users. Given the high level of function and experience using a microprocessor knee, there is no concern that a powered ankle-foot device connected to a prosthetic knee is a "potentially dangerous mechanical setup". In fact, the institutional review board for this study has deemed the study "minimal risk". Additionally, prosthesis user's muscles are not complacent with passive devices and will not be complacent with the addition of powered push-off. Second, prior to award of this grant, the investigators conducted a pilot trial to evaluate the safety of using a powered ankle-foot device in conjunction with a microprocessor knee for individuals with transfemoral limb loss. The study found no safety concerns for any users. No falls or stumbles were reported, and biomechanical outcomes showed improved gait symmetry (see Preliminary Studies). Additionally, the study provided recommendations on best practices for programming the ankle and knee units for optimal outcomes. Lastly, any participant who does not score a minimum of 33 on the AMPPRO or is deemed unsafe by the study prosthetist or physical therapist will not advance in the study (i.e., they will be unable to participate). These participants will be offered guided rehabilitation at the Department of Physical Medicine and Rehabilitation to enhance their use of their prosthetic device until they are able to meet the minimum standard of function. Once this baseline outcome is achieved, participation can occur. The process of guided rehabilitation may take multiple visits. Participants will not be able to start the study until they meet the functional criteria to enroll in the study.

*2) There is no consensus (supportive evidence) on the best PT practice in this area—the intervention is extrapolated from previous research/experience.*

**RESPONSE:** This study will be the first to report evidence regarding a physical therapy intervention for individuals with transfemoral limb loss who use a powered ankle-foot device. The physical therapy program is based on the significant experience of the physical therapists who have worked with this population for a combined 25 years. The results will be used to help determine appropriate timing and dosage of physical therapy, as well as to refine the program. The data and results will be used to refine the physical therapy program so that it can be effectively translated into clinical care.

*3) The preference and/or weight of the power ankle for the population of the study may influence results—especially with the dysvascular population.*

**RESPONSE:** This data (weight and preference) will be collected as part of subjective outcome measures. These variables will be assessed to determine their influence on results. If significant, these parameters can be controlled for during regression analyses.

*4) The information collected as part of Aims 1 through 3 will be useful to develop guidelines, but no information is provided to detail how the data will be translated into rehabilitation guidelines.*

**RESPONSE:** This study will be the first to correlate biomechanical, functional, pain, and neurocognitive and cognitive load variables with overall efficacy of using a powered ankle-foot device. As stated in the objective, this information will be evaluated and used to develop *preliminary* rehabilitation guidelines for a powered ankle-foot device to minimize gait imbalances and maximize function, as well as establish preliminary guidelines for powered ankle-foot prosthetic prescription. No other studies (to-date) have attempted to develop these guidelines for rehabilitation or prescription. Given the study design, it is well-suited to determine which parameters most closely correlate with overall efficacy to maximize function.

*5) Another weakness comes from the small sample size (30 participants).*

**RESPONSE:** For limb loss-related studies, a sample size of 30 participants with transfemoral limb loss is commensurate with other published studies in the literature and is considered a large sample size for this population. The power analysis indicates that a sample of 30 participants will provide sufficient power (>80% for variables of interest) to detect a true effect and will not be distorted by random or systematic error.

*6) The length of follow-up is also not well supported. The proposed study will test patients at baseline, after 4 weeks, and after 8 weeks. There is a good chance that patients are still adjusting to their new limb and that further normalization between the 2 patient groups would continue past the 8-week mark.*

**RESPONSE:** To-date, there is no consensus on the acclimation period for adjusting to a prosthetic device, specifically for a powered ankle-foot device. The length of physical therapy was based on recommendations from the American Physical Therapy Association. As such, 4 weeks (the recommended dosage) would allow for approximately 8 sessions of PT. The investigators considered it critical to assess outcomes directly following the physical therapy program, as well as at the 4 week follow up. Additionally, the timing of follow-up visits was based on a combination of clinical guidelines/consensus and reduction of participant burden. However, by comparing individuals who receive device-specific PT to those who receive the standard of care, we will likely be able to isolate the effects of the physical therapy program on functional and subjective outcomes. This information can be used to determine if this physical therapy program "speeds" up acclimation or if additional therapy may be needed. If it is determined that further normalization would continue past the 8-week mark, this information will be critical for clinical care (for those who are prescribed a powered ankle-foot device), as well as the design of future research. As such, the timing of this study will be critical in assessing the dosage and duration of care needed for this population.

*7) It is unclear if the device-specific rehabilitation will be instructed by 1 person at a single institution or different people at the institutions.*

**RESPONSE:** There will be a physical therapist at each site conducting the device-specific physical therapy program. Each therapist will undergo extensive training on the protocol to reduce variability of implementing the protocol at each site. Additionally, the program is based on meeting device-specific and PT goals for advancement through the program. Each participant who undergoes physical therapy will be required to meet the goals of each level prior to advancing. Therefore, each participant will meet the same goals in the study upon completion of the program. This will help reduce variability between sites.

*8) Additional concerns exist about the large weight of the foot, and this might be difficult for older and/or weaker patients.*

**RESPONSE:** The powered ankle-foot device is designed to be the average weight of an adult ankle-foot complex. While it is heavier than other prosthetic devices, the powered push-off reduces the effect of added weight on the participant. As previously described, participants in study will be advanced community ambulators (based on meeting a minimum standard of function for inclusion). As such, the added weight will not likely be difficult for participants who enroll in this study.
